# Air sampling to assess potential generation of aerosolized viable bacteria during flow cytometric analysis of unfixed bacterial suspensions

**DOI:** 10.12688/gatesopenres.12759.2

**Published:** 2018-02-27

**Authors:** Christine F Carson, Timothy JJ Inglis

**Affiliations:** 1 School of Biomedical Sciences (M504), Faculty of Health and Medical Sciences, University of Western Australia, Crawley, WA, Australia; 2 School of Medicine, Faculty of Health and Medical Sciences, University of Western Australia, Crawley, WA, Australia; 3 Department of Microbiology, PathWest Laboratory Medicine WA, Queen Elizabeth II Medical Centre, Nedlands, WA, Australia

**Keywords:** biosafety, laboratory biocontainment, bacteria, risk assessment, flow cytometer

## Abstract

This study investigated aerosolized viable bacteria in a university research laboratory during operation of an acoustic-assisted flow cytometer for antimicrobial susceptibility testing by sampling room air before, during and after flow cytometer use. The aim was to assess the risk associated with use of an acoustic-assisted flow cytometer analyzing unfixed bacterial suspensions. Air sampling in a nearby clinical laboratory was conducted during the same period to provide context for the existing background of microorganisms that would be detected in the air. The three species of bacteria undergoing analysis by flow cytometer in the research laboratory were *Klebsiella pneumoniae, Burkholderia thailandensis *and *Streptococcus pneumoniae*. None of these was detected from multiple 1000 L air samples acquired in the research laboratory environment. The main cultured bacteria in both locations were skin commensal and environmental bacteria, presumed to have been disturbed or dispersed in laboratory air by personnel movements during routine laboratory activities. The concentrations of bacteria detected in research laboratory air samples were reduced after interventional cleaning measures were introduced and were lower than those in the diagnostic clinical microbiology laboratory. We conclude that our flow cytometric analyses of unfixed suspensions of *K. pneumoniae, B. thailandensis *and *S. pneumoniae* do not pose a risk to cytometer operators or other personnel in the laboratory but caution against extrapolation of our results to other bacteria and/or different flow cytometric experimental procedures.

## Introduction

Flow cytometry techniques have been used to analyze bacteria for several decades ^1, 2^, and for assessing the effects of antimicrobial agents since the 1980s ^3– 5^. The bacteria analyzed then included species that can be safely handled on an open bench in a suitably equipped microbiology laboratory, providing a series of standard biosafety procedures are adhered to ^6^. Concerns about laboratory biosafety and containment increased after 2001 ^7, 8^ and led to higher physical containment levels for select biothreat agents and some bacterial species such as *Neisseria meningitidis,* prone to transmission by aerosols generated during laboratory procedures ^9, 10^. Given these concerns about bioaerosol transmission risks, it is not surprising that standards for bioaerosol risk assessment and mitigation have been recommended for fluorescence-activated cell sorting protocols ^11, 12^. Our use of flow cytometry was not for cell sorting, but for a less aerosol-prone cellular analysis. We commenced use of our analytic flow cytometer in a physical containment level two laboratory while developing a flow cytometry-assisted antimicrobial susceptibility test (FAST) assay method with *Klebsiella pneumoniae* ^13^. Though the cytometer we used had no cell sorting function and therefore would not generally produce aerosols, ^14, 15^ we decided to conduct an assessment to confirm that viable bacteria are not aerosolized during use before progressing with any analysis of potentially more hazardous aerosol-transmitted species such as *Neisseria meningitidis, Mycobacterium tuberculosis* and *Burkholderia pseudomallei*.

This study aimed to detect aerosolized viable bacteria during operation of an acoustic-assisted flow cytometer and to compare the detected bacteria with those cultured from air samples collected in the same laboratory space when the cytometer was not in use, and in a diagnostic clinical microbiology laboratory.

## Methods

**Laboratory locations.** Two laboratory locations in adjacent buildings were used. One was a university research laboratory approximately 54.7 m ^2^ and 145.5 m ^3^ equipped with two acoustic-assisted flow cytometers, and two class two biosafety cabinets, peripheral benches and one central bench. One of the flow cytometers is housed and used in a biosafety cabinet while the other one, the focus of this study, is on the open bench. The other location was a large, open plan clinical laboratory microbiology laboratory approximately 400.5 m ^2^ and 1081.4 m ^3^ operating a range of high throughput bacteriology procedures serving an on-campus 700 bed teaching hospital and an extensive regional hospital network. Notably, the clinical laboratory does not have an acoustic-assisted flow cytometer. Both laboratories were air-conditioned and equipped with high efficiency particulate air filtration on external air outlets. Detailed information about the number of complete air exchanges per hour was not available for either location.

The clinical laboratory was included for comparative purposes. While the study aimed to determine if aerosolized viable target bacteria ( *Burkholderia* *thailandensis*, *K. pneumoniae* and *Streptococcus pneumoniae*) could be detected in air in the research laboratory, the likelihood was that other microorganisms, commonly present in indoor air, would be detected. Published data regarding the range and concentration of bacteria present in air in microbiology laboratories, either research or clinical, are scarce. Without data for comparison, data on the viable bacteria detected in air samples from the research laboratory would be entirely without context. Work practices in the clinical laboratory are designed to maintain a safe working environment and the background level of viable bacteria detected in clinical laboratory air could therefore serve as a proxy indicator of the acceptable level of viable bacteria in air in the research laboratory.

Since background microorganisms would be detected in the air and, in an *a priori* effort to put them in some context, we sampled a non-flow cytometer site within the same research laboratory (preparation bench) and a non-flow cytometry laboratory (clinical laboratory) that handles numerous human bacterial pathogens using standard diagnostic microbiological techniques and biosafety risk management procedures.

**Flow cytometer equipment and reagents.** An Attune NxT (ThermoFisher Scientific, Eugene, OR) acoustic-assisted flow cytometer was the focus of these air sampling investigations. The instrument uses acoustic radiation pressure to align particles in the center of a sample stream. This pre-focused stream is then injected into the sheath stream, which supplies an additional conventional hydrodynamic pressure to the sample. The instrument uses a sheath fluid branded “focusing fluid” that hydrodynamically focuses samples just prior to analysis. The focusing fluid is a proprietary mix of reagents including an unspecified broad spectrum antimicrobial agent (personal communication M. Ward, ThermoFisher, Eugene OR, USA).

**Workflow**. The air sampling study was conducted over a one month period in which the research laboratory was intensively used by up to nine people at one time during office hours, often with both flow cytometers in use at once. Each flow cytometer procedure was staffed by one cytometer operator and another scientist preparing bacterial suspensions for FAST and other cytometer assays, plus at least one of the above authors engaged in the air sampling procedure. The majority of flow cytometer experiments analyzed the *K. pneumoniae* isolates as previously reported ^13^. The other two species analyzed were *S. pneumoniae* and *B. thailandensis*. The clinical laboratory was staffed between 7.30am and 9pm by up to 20 people, with 1–3 people per side of each laboratory bench, conducting predominantly manual procedures with liquid and solid bacterial cultures. While clinical specimens were opened and blood cultures were subcultured in a class two biosafety cabinet, the majority of bacteriologic procedures were performed on the open bench in accordance with clinical laboratory safety policy ^6^.

**Air sampling**. Air sampling was performed with a compact impinger air sampler (MAS-100 Eco, EMD Millipore, Merck) that drew a defined volume of room air over an agar plate positioned in the air sampling unit under the air-permeable lid. Every air sampling in this study was performed at a rate of 100 L per minute for 10 minutes. This is the method used in clean room and operating theatre air quality assessment in government health settings in Western Australia. The device used an Anderson sampler principle to draw air at a constant rate pre-set by the operator onto a 90 mm diameter Petri dish containing agar culture medium, after an initial timed delay to allow the operator to withdraw from its vicinity. The lid surfaces were cleaned with 70% isopropyl alcohol before and after each use in accordance with the manufacturer’s instructions.

Two types of media were used; 5% horse blood agar (HBA) and MacConkey agar (MAC) (both supplied by PathWest Laboratory Medicine WA, Mt Claremont WA, Australia). HBA was included as a non-selective medium intended to allow the growth of any of the three target bacteria. MAC was included to minimize the growth of background non-target microorganisms (predicted to be mostly fungi or Gram positive bacteria) while still allowing the growth of *B. thailandensis* or *K. pneumoniae*. Samples taken on HBA while *S. pneumoniae* was being used in the research laboratory were incubated in the presence of 5% CO _2_. MAC plates were never incubated in CO _2_. All plates were incubated aerobically at 35°C. Colony forming units on both types of solid media were recorded after 24 hr incubation and expressed as CFU/1000 L air. Positive growth controls to confirm the ability of each of the three target bacteria to grow on the media under the chosen incubation conditions were performed.

Air was sampled for identical periods, volumes and locations, onto both agar media on each occasion. Air sampling was conducted before, during and at the end of a day to give a range of times reflecting different levels of research laboratory use and occupation. The same pattern of sampling was conducted for comparison in the clinical bacteriology laboratory. Two main sampling sites were used in the research laboratory (see sampling sites 1 and 2, Supplementary Figure 1). The second of these; sample site 2, was immediately adjacent to the sample introduction port of the flow cytometer between the cytometer and its operator. For comparison, sampling site 1 was on the preparation bench behind the operator approximately 2.5 m from the flow cytometer where 1 ml samples from bacterial cultures (generally 20–30 ml in trypticase soya broth or Mueller Hinton broth) were washed by centrifugation, mixed, diluted and further handled prior to analysis on the flow cytometer. In general, samples analyzed on the cytometer contained approximately 10 ^6^ bacteria/ml or less. During data acquisition for FAST assays, up to 12 samples were analyzed per bacterium. Sample acquisition halted after 1–3 minutes, and each FAST sample was acquired in technical triplicate.

Air sampling was conducted at two sites in the clinical laboratory; the open bench where wound swabs were plated directly onto agar, and adjacent to the rotary plating device used to inoculate agar plates with bacterial suspensions for disk diffusion antimicrobial susceptibility tests (see sites 4 and 5, Supplementary Figure 1).

In the research laboratory, further sets of air samples were collected on multiple occasions after a detergent and ethyl alcohol interventional clean of all laboratory surfaces, and replacement of laboratory gowns. Interventional cleaning was performed late on a Friday and post-clean air samples were acquired beginning the following Monday morning. Cleaning practices in the clinical laboratory were not changed during the course of this study.

**Equipment and bench surfaces.** On completion of repeated air sampling over one month, surface swabs were collected from the external flow cytometer housing above, below and around the flow sample introduction port, the nearby open bench and the preparation bench opposite before any interventional cleaning had occurred. Swabs (peel pouch Dryswab™ catalogue number MW112; Medical Wire and Equipment Company, Corsham, Wiltshire, England) were immersed in sterile 0.9% normal saline before rubbing vigorously over a 2.5 cm diameter circular area for 1.5 minutes in a spiral motion beginning at the centre and rotating the swab continuously. Swabs were inoculated onto HBA first and then MAC using opposite sides of the swab for each plate. Swabs were collected at each site on at least two occasions. Incubation conditions were as previously described for air samples. Colony forming units on both types of solid media were recorded after 24 hr incubation at 35°C and expressed as CFU/swab.

**Bacterial identification.** All bacteria growing on MAC were identified using the clinical bacteriology laboratory’s identification protocol. In short, after macroscopic examination and discretionary Gram stain analysis, definitive identification was by matrix-assisted laser desorption ionisation time of flight mass spectrometry (MALDI-TOF) from an extended mass spectrometer profile library applying thresholds of 1.80 and 2.00 as the acceptable lower limits for genus and species level identifications, respectively. Initial borderline identification was repeated on the same sample on the stainless steel target with repeated MALDI-TOF analysis. Potential clinically significant isolates known to be problematic by MALDI-TOF or showing borderline acceptable identification were then subject to supplementary methods such as substrate utilisation panels (e.g. API20E, BioMerieux, France). Microbial growth on HBA was frequently heavy making it difficult to isolate and identify all colonies. Consequently, only the six commonest colony types were identified plus any that resembled the three target bacteria being interrogated by flow cytometry. Due to the selective nature of MAC, fewer colonies occurred and attempts were made to identify all isolates growing on MAC.

**Statistical analysis.** Column statistics, Chi squared test and non-parametric tests (Mann-Whitney U test) were conducted with Prism statistical software (Prism 6.0, GraphPad, San Diego, CA).

## Results

Details of each sample including laboratory location, sampling site, date, time and laboratory activity status are shown in the supplementary data file. In the research laboratory, the bacteria isolated from over 50,000 L of air sampled before, during and after flow cytometer operation did not yield a single cultured colony of *K. pneumoniae, S. pneumoniae* or *B. thailandensis* ( Table 1A, Table 1B). None of these species was recovered from either medium during the air sampling period, nor were they recovered from surface swabs (data not shown) taken above, below and around the flow cytometer sample introduction port or other research laboratory surfaces. Bacterial suspensions grown on HBA for experiments conducted on the flow cytometer remained culturable throughout the air sampling period. Positive growth controls confirmed the ability of each of the three target bacteria to grow on the media under the chosen incubation conditions. The bacteria we isolated from air samples in both laboratories at 1-189 CFU/1000 L air were predominantly commensal skin organisms such as coagulase-negative staphylococci and micrococci. The Gram negative bacteria we isolated on either medium by air sampling in both laboratories were environmental species. In the clinical laboratory, Gram negative bacteria were not amongst the six most common colony types detected on HBA. They were detected on MAC at concentrations of 1-5 CFU/1000 L air, much lower than the concentrations of Gram positive bacteria detected on HBA.

**Table 1A.** Identities of the commonest bacterial species isolated on blood agar ^†^ from 1000 L air samples, arranged by laboratory location. ^†^sampling onto HBA frequently yielded plates crowded with microbial growth. Only the six commonest colony types were identified plus any that resembled the three target bacteria ^‡^results from both sampling sites in the clinical laboratory were pooled for this table ****

		Bacteria identified from blood agar		
Laboratory location	Sampling site	Gram positive cocci	Gram negative bacilli	Gram positive bacilli
Research laboratory	Sample introduction port of the acoustic-assisted flow cytometer	*Staphylococcus* *caprae,* *S. epidermidis,* *S. haemolyticus,* *S. hominis,* *S. saprophyticus,* *S. warneri,* *Micrococcus luteus,* *Massilia timonae*	*Acinetobacter lwoffi,* *Pseudomonas oryzihabitans,* *Pantoea agglomerans*	*Bacillus licheniformis,* *B. megaterium*
	Preparation bench	*S. epidermidis,* *S. warneri,* *S. caprae,* *S. xylosus,* *S.equis,* *S. haemolyticus,* *S. hominis,* *S. saprophyticus,* *M. luteus*	*P. oryzihabitans,* *P. luteola*	*Agrobacterium* *radiobacter*
Clinical laboratory	Open benches ^‡^	*S. epidermidis,* *S. haemolyticus,* *S. saprophyticus,* *S. warneri,* *S. caprae,* *M. luteus*	(not present among identified bacteria) ^†^	(not present among identified bacteria)

**Table 1B.** Incidence of isolation of all Gram negative bacterial species sampled onto MacConkey agar and identified in 1000 L air samples according to laboratory location. ^‡^results from both sampling sites in the clinical laboratory were pooled for this table

	Laboratory location and sampling site		
	Research laboratory		Clinical laboratory
Gram negative bacteria identified from MacConkey agar	Acoustic-assisted flow cytometer	Preparation bench	Open benches ^‡^
*Pseudomonas luteola*	0	1	3
*P. oryzihabitans*	4	3	0
Unidentifiable	1	0	3
No bacterial growth	17	14	13

**Figure 1.**
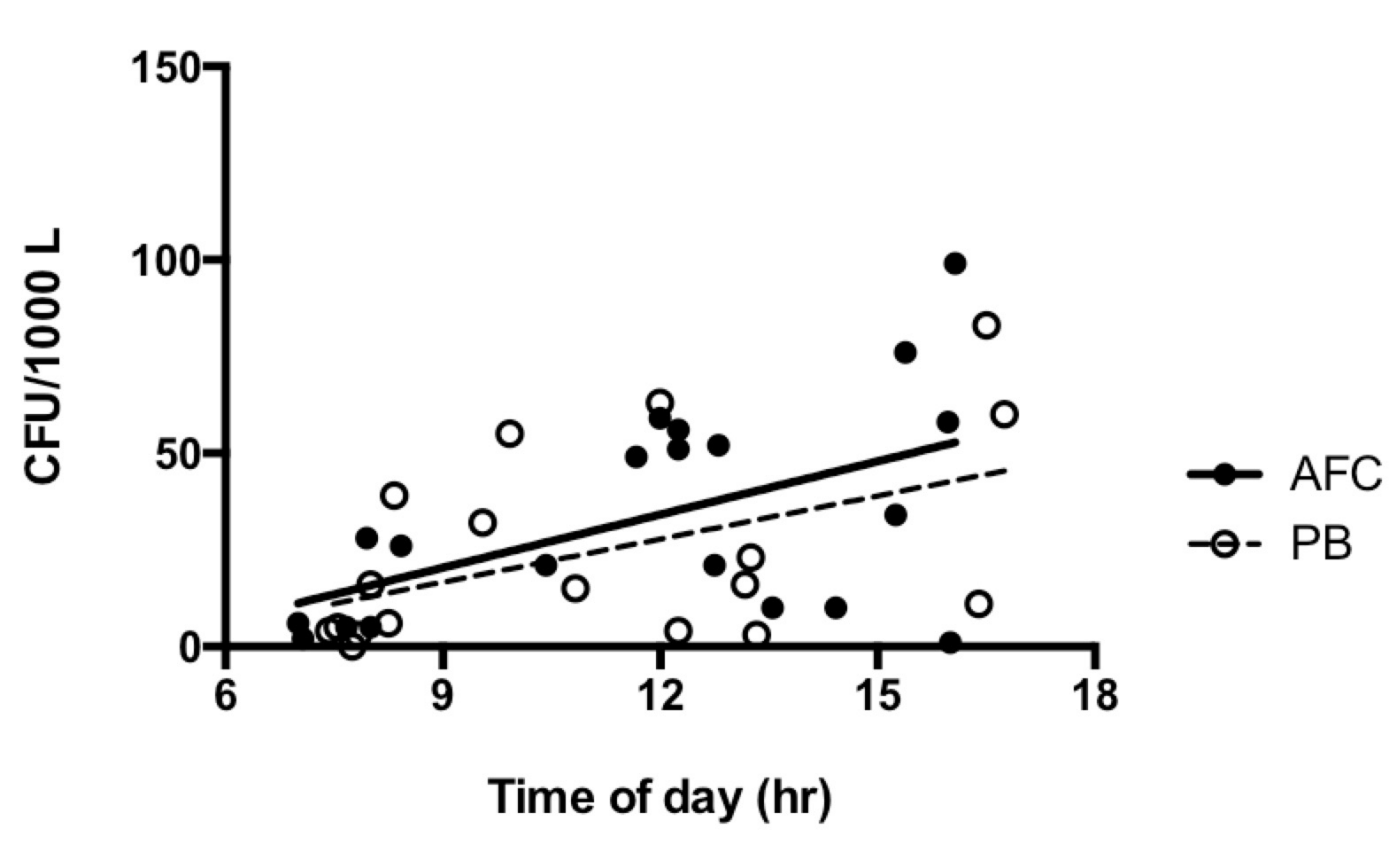
Airborne bacterial count detected on blood agar increases in the research laboratory over the course of the day, both beside the acoustic-assisted flow cytometer (AFC) and at the preparation bench (PB) (respective regression lines shown).

**Figure 2.**
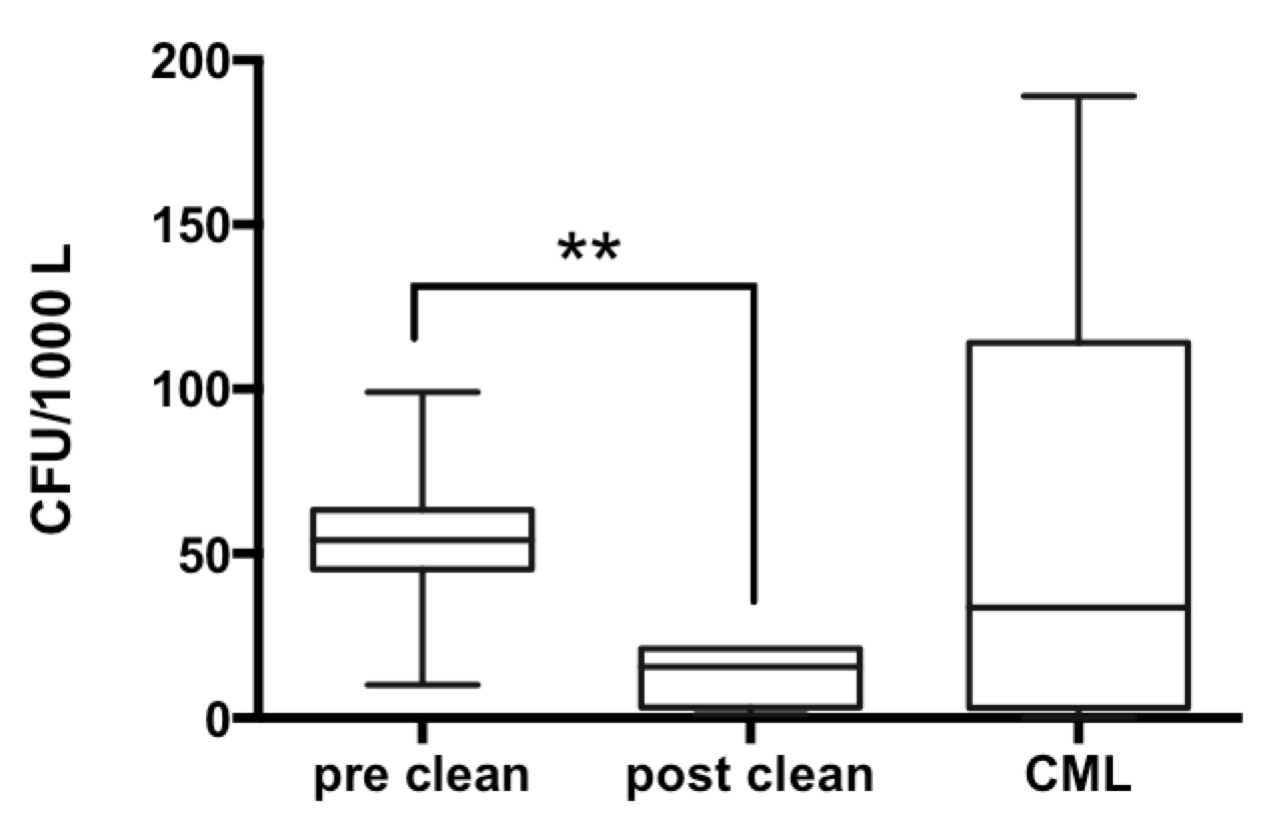
Airborne bacterial count close to acoustic flow cytometer sample introduction port during use, before (pre clean) and after research laboratory cleaning (post clean), compared with samples from the clinical microbiology laboratory (CML) where no interventional cleaning was undertaken. Pre clean research laboratory and post-clean median counts in close proximity to flow cytometer sample introduction port = 54 and 15.5 CFU/ 1000 L, U = 2.5, **p = 0.0090.

Total bacterial air counts increased during the day from <10 to 80–90 CFU/1000 L air both adjacent to the flow cytometer and at the nearby preparation bench ( Figure 1). Airborne bacterial counts (CFU/1000 L air) in the clinical bacteriology laboratory spanned a wider range of values but not a significantly different distribution ( Table 2). There was a significant fall in bacterial counts from air samples collected adjacent to the flow cytometer (54 CFU/1000 L air falling to 15.5 CFU/1000 L air) after interventional cleaning of research laboratory surfaces and replacement of gowns ( Figure 2).

**Table 2.** Bacterial counts (CFU/ 1000 L air) recovered from air sampled onto blood agar in the Research Laboratory or the Clinical Laboratory. ^†^‘In use’ refers to cytometer in operation for bacterial analysis. ^‡^results from both sampling sites in the clinical laboratory were pooled for this table

Laboratory location	Sampling site	Number of samples	Number of CFU/1000 L air on blood agar		
			Lower limit	Median	Upper limit
Research laboratory	Acoustic flow cytometer, not in use ^†^	9	0	5	85
	Acoustic flow cytometer, in use	10	10	54	99
	Acoustic flow cytometer, post clean	4	1	15.5	21
	Preparation bench	20	0	15.5	83
Clinical laboratory	Open benches ^‡^	14	0	33.5	189
All locations	All sites	57	0	21	189

## Discussion

The stated aim of the present study was to assess the potential risk of personnel exposure to viable bacteria aerosolized during analysis of unfixed bacterial suspensions with an acoustic-assisted flow cytometer, and taking note of current concepts of laboratory safety ^14, 15^, determine mitigation measures to reduce any risk posed by the future introduction of this flow cytometer method into a clinical laboratory. The absence in research laboratory air samples of any of the three bacterial species analyzed with the flow cytometer over a month of intensive laboratory work indicates that these three species represent a low aerosolization risk during the procedures we currently use. None of the analyses involve fluorescence-activated cell sorting, a process known to potentially generate bioaerosols ^11, 12, 16^, and for which standard methods of detection have been developed ^11^. The absence of any of these three species on the external surfaces of the flow cytometer is further evidence that the cytometer sample introduction port does not generate bacterial aerosols and that basic procedures of wiping surfaces with 70% ethyl alcohol are sufficient to adequately maintain a safe working environment. While our data applies only to the three species we assessed and does not necessarily predict the likelihood of other bacteria becoming airborne during flow cytometer procedures, it is likely that the occupational risks of handling other bacterial species with the same physical properties can be assessed by this approach. Clinical laboratories are subject to robust occupational health and safety practices in order to render them safe places to work. By comparing the viable bacterial content of the two laboratories we aimed to establish that the use of the flow cytometer for bacterial analysis would not contribute an additional bacterial load when this equipment is used in future in a clinical service laboratory.

The dominance of skin commensal and environmental species, which has been reported before ^17, 18^, raises wider issues of industrial hygiene which have not been actively investigated in clinical bacteriology laboratories handling class two pathogens. Our air sampling data indicate that viable airborne bacteria are common and may reach high concentrations of colony forming units during peak laboratory working hours. These data do not distinguish between skin bacteria shed by laboratory staff and disturbance of those same species resting on inanimate surfaces, as these issues were beyond the scope of our investigation. Nevertheless, the reduction in airborne bacterial counts documented after a thorough cleaning of research laboratory surfaces and replacement of laboratory gowns suggests that dust particles may be a contributory factor. These sources may be a lesser concern in a clinical bacteriology laboratory, but in a research laboratory developing novel methods of analyzing bacteria, airborne bacteria represent a potential source of media and equipment contamination that need to be brought under control. Contamination of flow cytometer focusing, wash or other fluids, even with non-biological particles, is a source of background noise and needs to be avoided for optimal results. For this reason, bacterial flow cytometer analyses demand some of the contamination control discipline exercised in molecular diagnostic laboratories. The contamination control measures incorporated into FAST procedures include 0.1 μm filtration of all fluids, flow cytometer analysis of suspending fluids for background particulate noise prior to bacterial analyses, sodium hypochlorite treatment of all flow cytometer effluent, and housing one of the acoustic flow cytometers inside a non-operating class two biosafety cabinet. Use of the non-operating cabinet gives the benefit of a physical barrier between the sample introduction port and the operator. Unfortunately, coarse vibration associated with safety cabinet operation may interfere with the accuracy of flow cytometer data capture. Housing the flow cytometer inside a static bio-bubble of suitable biocontainment level will cause less interference from vibration, when analyzing bacterial species requiring a higher biosafety containment level.

This study showed that use of an acoustic-assisted flow cytometer for bacterial analysis over extended periods did not contribute detectable concentrations of test bacteria to the population of bacteria we cultured from air samples collected in a research laboratory environment. Further, the majority of bacteria cultured were skin commensal and environmental bacteria, presumed to have been shed and dispersed or distributed in laboratory air by personnel movements during routine laboratory operation. The concentrations of airborne bacteria detected in the research laboratory were comparable with those detected in a nearby clinical laboratory on the same biomedical campus and were significantly reduced after cleaning measures were introduced in the research laboratory.

The limitations of this study include the relatively small number of samples, the short duration of the observations collected over the course of about one month and the paucity of data available for comparison.

We undertook this work as part of a risk assessment of the hazards posed by analysing unfixed bacteria by flow cytometric methods. None of the bacteria being investigated by flow cytometry were detected in air samples, irrespective of whether the cytometers were in use. Furthermore, the levels of airborne bacteria detected in the research laboratory were lower than those detected in a large clinical bacteriology laboratory located in an adjacent building on the same campus. We conclude that our flow cytometric analyses of unfixed suspensions of *K. pneumoniae, B. thailandensis* and *S. pneumoniae* do not pose a risk to cytometer operators or other personnel in the laboratory but caution against extrapolation of our results to other bacteria and/or different flow cytometric experimental procedures.

## Data availability

The data supporting the findings reported in this study have been uploaded to OSF: osf.io/z8uka ^19^.

**Dataset 1: FAST project bacterial air sampling.** Quantitative air sampling data, times, locations, corresponding laboratory activities and qualitative bacteriologic identification results.

**Datasets 2 – 6: FAST air sampling Table 1A, Table 1B, and Table 2, and Figure 1 and Figure 2.** Analysis of bacterial air sampling during use of acoustic-assisted flow cytometer for bacterial analysis and control settings.

Data are available under the terms of the Creative Commons Zero “No rights reserved” data waiver (CC0 1.0 Public domain dedication).

## Supplementary material

**Supplementary Figure 1.** Floor plan of both laboratories used in this study drawn to the same relative scale: A. research laboratory and B. clinical laboratory. Numbered locations: 1. preparation bench, 2. acoustic-assisted flow cytometer, 3. additional bench location used for air sampling, 4. clinical laboratory culture examination bench, 5. Clinical laboratory antimicrobial susceptibility test bench. Biosafety cabinets indicated by diagonal hatched boxes.
