# Supplementary figures and images for: Air sampling to assess potential generation of aerosolized viable bacteria during flow cytometric analysis of unfixed bacterial suspensions
Source: Gates Open Res. 2018 Feb 27;1:2. Originally published 2017 Nov 6. [Version 2] doi: 10.12688/gatesopenres.12759.2 (PMC5873458; doi:10.12688/gatesopenres.12759.2)

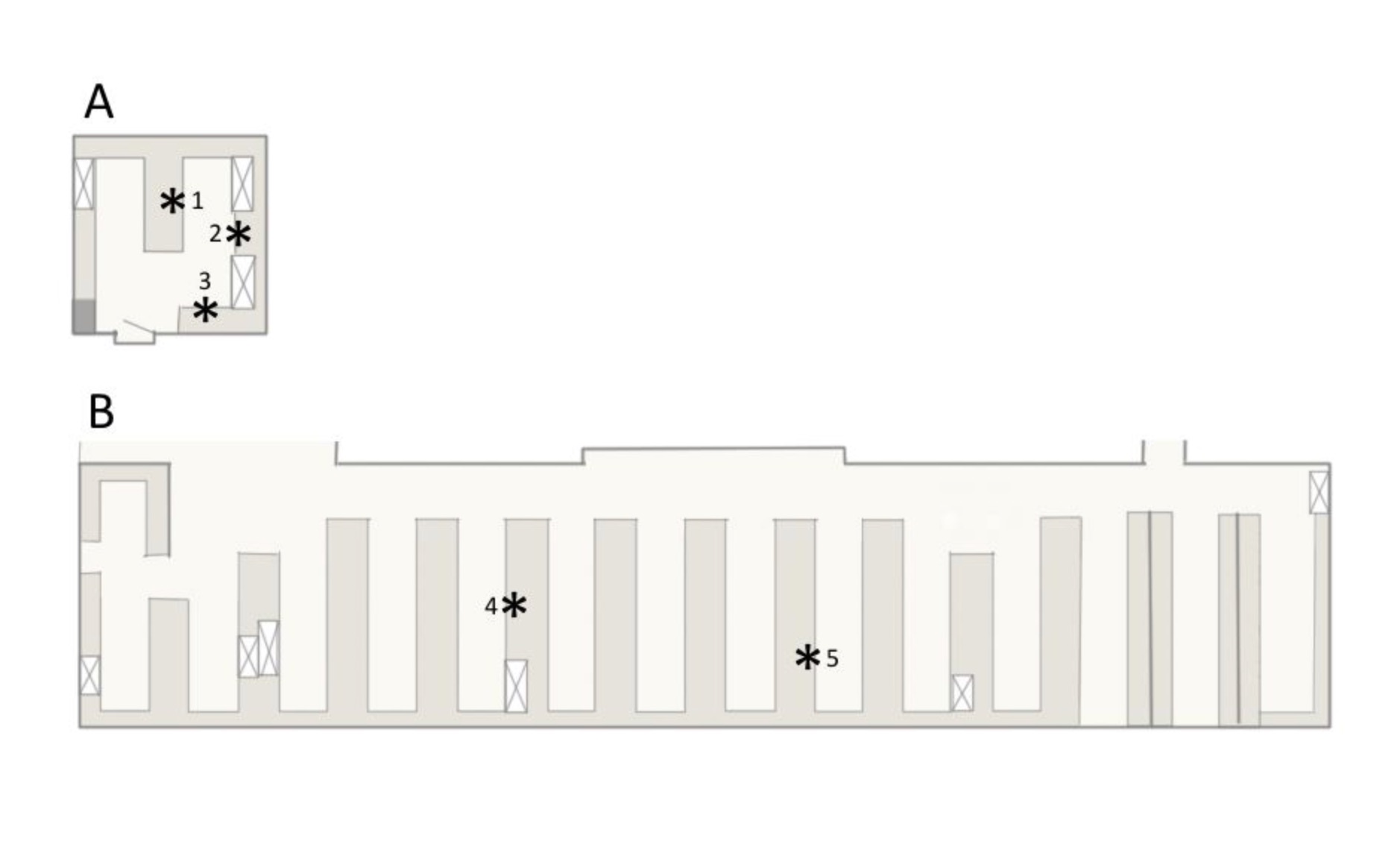

Supplement: Supplementary file 1 [file gatesopenres-1-13863-s0000.tgz › 329adf5f-76ed-4fb9-984f-2e1439e1726f.jpg]
