# Supplementary material for: Air sampling to assess potential generation of aerosolized viable bacteria during flow cytometric analysis of unfixed bacterial suspensions
Source: Gates Open Res. 2018 Feb 27;1:2. Originally published 2017 Nov 6. [Version 2] doi: 10.12688/gatesopenres.12759.2 (PMC5873458; doi:10.12688/gatesopenres.12759.2)
Supplement: Peer review history

# Peer review: Referee response for version 2

**DOI:** 10.21956/gatesopenres.13863.r26278

**Published:** 2018-04-25

**Authors:** Coralie L. Guerin, Lea Guyonnet

**Affiliations:** 1 National Cytometry Platform, Department of Infection and Immunity, Luxembourg Institute of Health, Strassen, Luxembourg; 2 Luxembourg Institute of Health, Strassen, Luxembourg

We would like to thank the authors for taking in consideration our comments in their new version.

 In this second version, the introduction still remains unclear whether the authors are focussing on “natural” aerosols generated by laboratory practices (ref 9 and 10) or aerosols generated by hydrofocussed flow cytometer (ref 11 and 12). In order to point out the exact purpose of this study and to avoid misunderstanding, the authors should clarify more. Definitions for each of the phenomenom should be given.

 In our first reviewing, the authors were asked to provide positives controls. Indeed, there is a line wich was added in the text mentioning that positive controls were performed. In order to validate this work, authors need to provide data of those positives controls.

We have read this submission. We believe that we have an appropriate level of expertise to confirm that it is of an acceptable scientific standard, however we have significant reservations, as outlined above.

# Peer review: Referee response for version 2

**DOI:** 10.21956/gatesopenres.13863.r26279

**Published:** 2018-03-28

**Authors:** Peter A. Lopez

**Affiliations:** 1 Department of Pathology, New York University School of Medicine, New York, NY, USA

The basic premise of the study, to show that in routine operation the Attune flow cytometer does not release bacteria of the type specified in the environment, seems to be addressed, although sub-optimally in this reviewer's opinion.The author's observation could still be attributed to anti-microbials in the Attune operating buffer.

 The authors use of the clinical laboratory as a control is still questioned.

 Sadly, Supplementary Figure 1 does little to clarify the laboratory setting for the reader.. Even though lab area is described in the text, no scale is included in the lab layout drawing. The reader can not discerne if there are open areas or walls of shelving that might impede air flow. In addition, information about the facility airflow (air register placement in relation to the instrument, total room air changes per hour, filtration of air) were not found by this reviewer.

I have read this submission. I believe that I have an appropriate level of expertise to confirm that it is of an acceptable scientific standard, however I have significant reservations, as outlined above.

# Peer review: Referee response for version 2

**DOI:** 10.21956/gatesopenres.13863.r26277

**Published:** 2018-03-20

**Authors:** Tor Monsen

**Affiliations:** 1 Department of Clinical Microbiology, Umeå University, Umeå, Sweden

The manuscript has been revised accordingly and has improved. The authors have added significant information that has improved the quality and message of the present manuscript. However i do lack information regarding the number of staff / m3 (or m2) both in for the research laboratory and the research laboratory. The number of bacteria in air correlated to the number of staff /m3 (or m2). In this study one can assume that the ventilation system is "nearly the same", hence the number of bacteria in air is correlated to the number of staff/m3 (or m2). The estimated numbers/m3 (or m2) are important to interpret the results of Figure 2.

 Beside this comment I do not have further comments to the present manuscript.

 I do recommend the article to be accepted after addition of the requested figures.

 Recommended articles to the authors

https://www.tandfonline.com/doi/pdf/10.3155/1047-3289.61.7.732

https://www.ncbi.nlm.nih.gov/pmc/articles/PMC3444341/ 

http://images.biomedsearch.com/25006349/1754-9493-8-27.pdf?AWSAccessKeyId=AKIAIBOKHYOLP4MBMRGQ&Expires=1521590400&Signature=kAirg%2B1ZaQ6pet6yrDiKwruiTJk%3D

I have read this submission. I believe that I have an appropriate level of expertise to confirm that it is of an acceptable scientific standard.

# Peer review: Referee response for version 1

**DOI:** 10.21956/gatesopenres.13819.r26119

**Published:** 2017-12-08

**Authors:** Peter A. Lopez

**Affiliations:** 1 Department of Pathology, New York University School of Medicine, New York, NY, USA

Thank you for the opportunity to participate in the review of the manuscript " Air sampling during flow cytometric analysis of unfixed bacterial suspensions; a risk assessment "

 I believe the authors were trying to assess the possibility of aerosol generation from bacterial samples run on an acoustically-focused flow cytometric amalyzer. It is not clear if the intent of the paper was to document potential aerosol, or aerosolized viable microbes. This very important distinction would add to the paper's reach. Regardless, their testing methods were not as rigorous as previous publications of a similar nature ( which they did include in their references ( their reference 11 and 12 ), and only confirmed information already available -- that analytical cytometers do not generate aerosol during routine operation ).

 Of note, the authors did not include pertinent references that point to the lack of aerosol generation by analytical flow cytometers ( see citations 1&2, added in this review ).

 While additional comments are made in the body of this review, one critical piece of information that is missing in this study revolves around the fact that flow cytometers, including the Attune, add a buffer to the sample during the analysis. Some buffers, such as the FACSflow buffer marketed by Beckton Dickinson, includes anti-microbial agents in the buffer. The authors should have inquired as to the potential anti-micobials that may be contained in the focusing buffer utilized in the Attune. Obviously if this buffer contains such additives the question of aerosolized viable microbes was not addressed.

 As the Attune line of instrumentation from Thermo Fisher utilizes a new technique for focusing cells as they pass through the instrument known as acoustic focusing, there may be concern that this new modality could introduce aerosolization of sample material. If the authors had utilized published methods of aerosol assessment as outlined in the papers that they referenced, this would have clearly added to the value and reproducible of this risk assessment study.

 The abstract itself is not clear and does not work work as a standalone summary of this work. In the abstract the authors they include mention of testing in a research laboratory, as well as a "nearby clinical laboratory" without noting why two different labs were being compared. The authors mention extensively that 1000 liter air samples were evaluated for airborne bacteria, with no rationale given for this volume of sampling. Readers are left to wonder if this is an industry-standard sample size, or alternatively,  if this may represent an under sampling.

 The study design and technical soundness of the work is questionable since the authors do not use previously published methods, do not provide any information regarding the bacterial concentration of samples run on the cytometer, or how many sample tubes were being run on the cytometer during the testing period. The authors did describe the lab workflow-- number of people in the lab, hours of operation, and types of tests, but this adds little to the information needed to reproduce this work, or to validate the impact of the results. A basic diagram of the lab, to include outlines of instrumentation and benches to scale, room dimensions, and including more detail of air-sampling locations, would have been very informative for this study.

 It is this reviewer's opinion that without this basic information this study could not be reproduced by a reader.

 Those skilled in the art might have concerns that the authors have different interpretations of widely used acronyms, using flourescence-assisted cell sorting rather that flourescence activated cell sorting (the original definition of FACS), and then building upon this interpretation in their definition of the FAST assay, a test which while referenced, could have been briefly described in more detail for those not familiar with this paper. Also HEPA fitration is defined in the paper as "high efficiency purified air", where the commonly utilized definition is either High-Efficiancy Particulate Air, or High-Efficiency Particulate Arrestor. Lastly, the authors continue this trend to introduce AFC for acoustic-enhanced flow cytometer, an acronym not even used by the instrument manufacturer. While minor, these lapses may raise questions in the reader's mind.

 While the conclusions drawn may be accurate-- that the authors saw no bacteria becoming airborne as a result of acoustically-focused flow cytometry in their assay, their methods can be challenged and would be difficult to reproduce.

 In light of the points made above, I am sorry to suggest that the current study in its current form should be rejected

I have read this submission. I believe that I have an appropriate level of expertise to state that I do not consider it to be of an acceptable scientific standard, for reasons outlined above.

## References

[1] MillerJMAstlesRBaszlerTChapinKCareyRGarciaLGrayLLaroneDPentellaMPollockAShapiroDSWeirichEWiedbraukDBiosafety Blue Ribbon PanelCenters for Disease Control and Prevention CDC: Guidelines for safe work practices in human and animal medical diagnostic laboratories. Recommendations of a CDC-convened, Biosafety Blue Ribbon Panel. *MMWR Suppl*.2012;61(1) :1-102 22217667

[2] SewellD.LBoveK.ECallihanD.R: Protection of Laboratory Workers from Occupationally Acquired Infections. *Approved Guideline—Third Edition (M29-A3)*.2005;

# Response

**Published:** 2018-02-05

**Authors:** Timothy Inglis

**Reviewer**: I believe the authors were trying to assess the possibility of aerosol generation from bacterial samples run on an acoustically-focused flow cytometric amalyzer. It is not clear if the intent of the paper was to document potential aerosol, or aerosolized viable microbes. This very important distinction would add to the paper's reach.

**Response**

- We have clarified text in the Title, Abstract, Introduction and Discussion to reflect that detection of viable aerosolized target bacteria before, during or after use of the acoustic-assisted flow cytometer was the aim of the study.

**Reviewer**: Regardless, their testing methods were not as rigorous as previous publications of a similar nature (which they did include in their references ( their reference 11 and 12 ), and only confirmed information already available -- that analytical cytometers do not generate aerosol during routine operation ).

- This was a very practical investigation designed to investigate if there was a risk of exposure to aerosolized test bacteria for personnel using the flow cytometer.

**Reviewer:** Of note, the authors did not include pertinent references that point to the lack of aerosol generation by analytical flow cytometers ( see citations 1&2, added in this review ).

- These references suggested by the reviewer are relevant and have been inserted: Sewell et al. and Miller et al., in the second last paragraph of the Introduction.

**Reviewer: **While additional comments are made in the body of this review, one critical piece of information that is missing in this study revolves around the fact that flow cytometers, including the Attune, add a buffer to the sample during the analysis. Some buffers, such as the FACSflow buffer marketed by Beckton Dickinson, includes anti-microbial agents in the buffer. The authors should have inquired as to the potential anti-micobials that may be contained in the focusing buffer utilized in the Attune. Obviously if this buffer contains such additives the question of aerosolized viable microbes was not addressed.

 

- Inclusion of an antimicrobial agent in the Attune Focusing Fluid has been clarified in the “Flow cytometer equipment and reagents” Methods section. Advice from the manufacturer is that some of the first part of the sample may mix with the sheath fluid but that the bulk of the sample, centered by the focusing forces, would only be exposed to the agent through diffusion. The window of opportunity during which contact between the sample and the sheath fluid could occur by diffusion is between the sample leaving the injector and reaching the laser.

**Reviewer:** As the Attune line of instrumentation from Thermo Fisher utilizes a new technique for focusing cells as they pass through the instrument known as acoustic focusing, there may be concern that this new modality could introduce aerosolization of sample material. If the authors had utilized published methods of aerosol assessment as outlined in the papers that they referenced, this would have clearly added to the value and reproducible of this risk assessment study.

- This was a practical assessment of the risk posed to personnel using the flow cytometer. Detection of viable aerosolized bacteria was attempted using the same method applied for clean room and operating theatre assessment in Western Australia. The manufacturer of the flow cytometer is confident that the instrument does not generate aerosols under normal working conditions.

**Reviewer: **The abstract itself is not clear and does not work work as a standalone summary of this work. In the abstract the authors they include mention of testing in a research laboratory, as well as a "nearby clinical laboratory" without noting why two different labs were being compared. The authors mention extensively that 1000 liter air samples were evaluated for airborne bacteria, with no rationale given for this volume of sampling. Readers are left to wonder if this is an industry-standard sample size, or alternatively,  if this may represent an under sampling.

- The Abstract has been substantially modified for clarity.
- The inclusion of the nearby clinical laboratory as a sampling site has been more clearly justified. See the additional paragraph under Methods, Laboratory Locations.
- The basis for 1000 L air sample size is provided in the Methods, Air Sampling section.

**Reviewer:** The study design and technical soundness of the work is questionable since the authors do not use previously published methods, do not provide any information regarding the bacterial concentration of samples run on the cytometer, or how many sample tubes were being run on the cytometer during the testing period. The authors did describe the lab workflow-- number of people in the lab, hours of operation, and types of tests, but this adds little to the information needed to reproduce this work, or to validate the impact of the results. A basic diagram of the lab, to include outlines of instrumentation and benches to scale, room dimensions, and including more detail of air-sampling locations, would have been very informative for this study.

 

- The range of bacterial concentrations of samples has been added to the text.
- A schematic of both laboratories has been added to the Supplementary information
- The approximate area and volume of each laboratory has been added to the text.

**Reviewer:** It is this reviewer's opinion that without this basic information this study could not be reproduced by a reader.

Those skilled in the art might have concerns that the authors have different interpretations of widely used acronyms, using flourescence-assisted cell sorting rather that flourescence activated cell sorting (the original definition of FACS), and then building upon this interpretation in their definition of the FAST assay, a test which while referenced, could have been briefly described in more detail for those not familiar with this paper. Also HEPA fitration is defined in the paper as "high efficiency purified air", where the commonly utilized definition is either High-Efficiancy Particulate Air, or High-Efficiency Particulate Arrestor. Lastly, the authors continue this trend to introduce AFC for acoustic-enhanced flow cytometer, an acronym not even used by the instrument manufacturer. While minor, these lapses may raise questions in the reader's mind.

 

- Abbreviations have been amended/eliminated and/or harmonized.

**Reviewer: **While the conclusions drawn may be accurate-- that the authors saw no bacteria becoming airborne as a result of acoustically-focused flow cytometry in their assay, their methods can be challenged and would be difficult to reproduce.

In light of the points made above, I am sorry to suggest that the current study in its current form should be rejected

**Response: **We thank the reviewer for their careful reading and commentary on this paper.

Major revision has been undertaken and we trust the manuscript is now acceptable for publication.

# Peer review: Referee response for version 1

**DOI:** 10.21956/gatesopenres.13819.r26113

**Published:** 2017-12-07

**Authors:** Coralie L. Guerin, Lea Guyonnet

**Affiliations:** 1 National Cytometry Platform, Department of Infection and Immunity, Luxembourg Institute of Health, Strassen, Luxembourg; 2 Luxembourg Institute of Health, Strassen, Luxembourg

In this study, the authors investigated the formation of bioaerosols while analyzing three species of bacteria with an acoustic-enhanced flow cytometer (AFC) in a research laboratory thus assessing the risk for the staff to be exposed to bacteria. The authors sampled room air during one month on daily basis before, during and after acoustic-enhanced flow cytometer use.

 There are some major comments concerning this manuscript.

1. Indeed, assess the risk of aerosol generation from samples loaded in cytometer is of interest in the field to evaluate and prevent potential transmission to operator. This risk of exposure is well known for hydrodynamic focusing cell sorter in which pressurization of the sample and jet in air system lead to high generation of aerosol. In the manuscript, authors are using an acoustic focusing cytometer where the sample is aspirated in a Hamilton syringe before being focused in the flow cell through ultrasound. Before showing absence of unfixed bacteria in aerosols around the AFC, authors should first test whether this instrument is generating aerosol by using glow germ for example.
2. The duration and frequencies of air sampling are not clearly mentioned. The authors should present a detailed timetable of the samplings indicating duration and frequencies.
3. The absence of CFU for air sampling/swabbing cannot be interpreted since positive and negative controls are missing. Experiments showing the ability of the three bacteria to grow on media used as positive control and experiments with culture media alone and ‘clean’ air sampling without bacteria loaded into the AFC as negative controls should be presented.    
4. Results from swabbing even negative need to be shown with appropriate controls.
5. In the discussion, comparison with hydrodynamic focused flow cytometer is missing. 

There are some minor comments concerning this manuscript.

1. Manuscript title does not reflect the aim of the study.
2. Regarding methods, the authors indicates that two AFC and two class II biosafety cabinets are presents in the research lab, do AFC are under those biosafety cabinets?
3. As comparison, the authors used a clinical laboratory that is not using AFC technique. In the manuscript, specification about size, layout and staff per m2 are missing. Specifications of both laboratory should be presented. 
4. Is the clinical laboratory equipped with air conditioning and/or HEPA filters? ie same air renewal per hour 
5. Concerning air sampling in the clinical laboratory, the authors mention that the sampling location was conducted at the open bench, was it near the operator? In the research lab, two sampling locations were used. Please comment the choice of having only one in the clinical laboratory. 
6. Which kind of media was used? FBA, HBA, BA are mentioned in the manuscript. If they are identical, harmonization should be done. If not, please explain differences. 

 

  In conclusion, the authors states that the risk for staff to be exposed to the analyzed bacteria is non-existent. The present study could be of interest but detailed information needed for full reproducibility and appropriate controls to be able to draw a conclusion are missing and do not allow conclusion regarding the study.

We have read this submission. We believe that we have an appropriate level of expertise to confirm that it is of an acceptable scientific standard, however we have significant reservations, as outlined above.

# Response

**Published:** 2018-02-05

**Authors:** Timothy Inglis

**Reviewer: **In this study, the authors investigated the formation of bioaerosols while analyzing three species of bacteria with an acoustic-enhanced flow cytometer (AFC) in a research laboratory thus assessing the risk for the staff to be exposed to bacteria. The authors sampled room air during one month on daily basis before, during and after acoustic-enhanced flow cytometer use.

There are some major comments concerning this manuscript.

1. Indeed, assess the risk of aerosol generation from samples loaded in cytometer is of interest in the field to evaluate and prevent potential transmission to operator. This risk of exposure is well known for hydrodynamic focusing cell sorter in which pressurization of the sample and jet in air system lead to high generation of aerosol. In the manuscript, authors are using an acoustic focusing cytometer where the sample is aspirated in a Hamilton syringe before being focused in the flow cell through ultrasound. Before showing absence of unfixed bacteria in aerosols around the AFC, authors should first test whether this instrument is generating aerosol by using glow germ for example.

- **Response**. The literature is replete with assertions that hydrodynamic flow cytometers without sorting capacity do not generate aerosols but empirical data are hard to find. All the publications containing data found with Google Scholar using the search phrase "Glo Germ" OR "Glo-Germ"  "flow cytometer" OR "flow cytometry" relate to cell sorting. We were unable to find any papers using Glo-Germ to evaluate aerosol generation on a hydrodynamic flow cytometer without cell sorting.
- The Glo Germ suggestion has some merit although it is important to consider that in terms of their aerosolization potential, all bacteria are not born equal. Numerous studies demonstrate that different bacteria are variably prone to aerosolization (Parker et al., Amer Rev Resp Dis 1983; Angenent et al., PNAS 2005; Gauthier-Levesque et al., BMC Res Notes 2016; Perrott et al., J Appl Microbiol 2017; Veillette et al., J Aerosol Sci 2018). We sought to determine if viable *K. pneumoniae, S. pneumoniae *or *B. thailandensis* from our assays were entering the air in and around the analytical instrument. Glo Germ are fluorescent 5-μm melamine copolymer resin beads used as bacterial facsimiles. Whether these adequately represent the aerosolization potential of different bacteria remains unclear.
- Our study was deliberately a very practical one designed to identify whether viable bacteria could be detected in the air around the acoustic flow cytometer sample introduction port and the operator during use. We sought to evaluate the risk these procedures posed to laboratory personnel. We appreciated that background microorganisms would be detected in the air and, in an *a priori* effort to put them in some context, sampled a non-flow cytometer site within the same laboratory (preparation bench) and a non-flow cytometry laboratory (clinical laboratory) that handles numerous human pathogens using standard diagnostic microbiological techniques.

**2. Reviewers**: The duration and frequencies of air sampling are not clearly mentioned. The authors should present a detailed timetable of the samplings indicating duration and frequencies.

- **Response**. The duration of sampling was identical in each run; 100 L per minute for 10 minutes.
- All the dates, times and frequencies of sampling are shown in the detailed data set uploaded to Open Science Forum at the doi provided with this article. 

**3. Reviewers**. The absence of CFU for air sampling/swabbing cannot be interpreted since positive and negative controls are missing. Experiments showing the ability of the three bacteria to grow on media used as positive control and experiments with culture media alone and ‘clean’ air sampling without bacteria loaded into the AFC as negative controls should be presented.

- **Response**. The following text has been added to the Air Sampling section to clarify these points:

Two types of media were used: 5% horse blood agar (HBA) and MacConkey agar (MAC) (both supplied by PathWest Laboratory Medicine WA, Mt Claremont WA, Australia). HBA was included as a non-selective medium intended to allow the growth of any of the three target bacteria. MAC was included to minimize the growth of background non-target microorganisms (predicted to be mostly fungi or Gram positive bacteria) while still allowing the growth of *B. thailandensis* or *K. pneumoniae*. Samples taken on HBA while *S. pneumoniae* was being used in the research laboratory were incubated in the presence of 5% CO _2_. MAC plates were never incubated in CO _2_. All plates were incubated aerobically at 35°C. Colony forming units on both types of solid media were recorded after 24 hr incubation and expressed as CFU/1000 L air. Positive growth controls to confirm the ability of each of the three target bacteria to grow on the media under the chosen incubation conditions were performed.

**4. Reviewers**. Results from swabbing even negative need to be shown with appropriate controls.

- **Response**. Positive growth controls demonstrated that the target bacteria were able to grow on the media. This information has been added to the text. All swab results are shown in the accompanying data set.

**5. Reviewers**. In the discussion, comparison with hydrodynamic focused flow cytometer is missing. 

- **Response**. Comparison with hydrodynamic systems is difficult since as mentioned above at point 1, while the literature is replete with assertions that hydrodynamic flow cytometers without sorting capacity do not generate aerosols, empirical data are hard to find.

**Reviewers**. There are some minor comments concerning this manuscript.

1.Manuscript title does not reflect the aim of the study.

- **Response**. The title has been amended to more accurately reflect the revised content.

2. Regarding methods, the authors indicates that two AFC and two class II biosafety cabinets are presents in the research lab, do AFC are under those biosafety cabinets?

- **Response**. This has been clarified in the manuscript:One of the flow cytometers is housed and used in a biosafety cabinet while the other one, the focus of this study, is on the open bench. 

**3. **As comparison, the authors used a clinical laboratory that is not using AFC technique. In the manuscript, specification about size, layout and staff per m2 are missing. Specifications of both laboratory should be presented. 

- **Response**. This information has been added to the manuscript. 

**4. **Is the clinical laboratory equipped with air conditioning and/or HEPA filters? ie same air renewal per hour 

- **Response**. No information was available about the air renewal per hour. The following has been added to the manuscript:Both laboratories were air-conditioned and equipped with high efficiency particulate air filtration on external air outlets. Detailed information about the number of complete air exchanges per hour was not available for either location.

**5. **Concerning air sampling in the clinical laboratory, the authors mention that the sampling location was conducted at the open bench, was it near the operator? In the research lab, two sampling locations were used. Please comment the choice of having only one in the clinical laboratory. 

- **Response**. Two sites were sampled in both laboratories. Both sampling sites in both labs were taken near the personnel. A third site was occasionally sampled in the research laboratory. Details of all the sample sites are shown in the supplementary material.

**6. **Which kind of media was used? FBA, HBA, BA are mentioned in the manuscript. If they are identical, harmonization should be done. If not, please explain differences. 

- **Response**. Abbreviations have been amended/eliminated and/or harmonized.

**Reviewers**. In conclusion, the authors states that the risk for staff to be exposed to the analyzed bacteria is non-existent. The present study could be of interest but detailed information needed for full reproducibility and appropriate controls to be able to draw a conclusion are missing and do not allow conclusion regarding the study.

We appreciate the reviewers' expert guidance on how to improve the presentation of our observations.

# Response

**Published:** 2018-02-05

**Authors:** Timothy Inglis

We have addressed the reviewers' comments and recommendations in an amended version of this manuscript, that more clearly states the purpose of our study, and the need to compare with a clinical laboratory environment. Additional data has been provided including a supplementary figure showing the respective laboratory floor plans. 

 We appreciate the reviewers' expert guidance on how to improve the presentation of our observations.

# Peer review: Referee response for version 1

**DOI:** 10.21956/gatesopenres.13819.r26069

**Published:** 2017-11-27

**Authors:** Tor Monsen

**Affiliations:** 1 Department of Clinical Microbiology, Umeå University, Umeå, Sweden

Many thanks for the opportunity to review the manuscript entitled “Air sampling during flow cytometric analysis of unfixed bacterial suspensions; a risk assessment” by Christine F Carson *et al*.

 The topic of the study is important in clinical laboratories regarding the risk for the staff to be exposed to harmful bacteria during their daily work. In this case risk for exposure to harmful bacteria during antimicrobial susceptibility test performed using a flow cell cytometric analysis. The method using “flow cytometric analysis” (FCA) is a relative new technique used in clinical laboratories and an expanding method I clinical microbiology.

 I do have some major and minor comment to the present manuscript that has to be taken in consideration prior to acceptance of the manuscript.

  

1. The title of the manuscript do not clearly describe the intention of the present study. I would suggest that the title adjusted or rewritten to the message of the the present study.
2. Abstract: lack of “Aim(s)” in the text. 
3. The authors includes a clinical laboratory (CL)  that do not use the FCA technique. However the bacterial concentration in air is measured and compared with that in the research laboratory using the FCA technique. However the design of the two laboratories (research- and clinical laboratory) are very different in construction and size (square meters) and if compared the number of staff/m2 should be presented.
  The clinical laboratory was included just to compare the distribution of bacteria in air with air the research laboratory which is of secondary interest as a risk assessment since the FCA instrument was not used in the clinical laboratory.
4. Regarding the research laboratory information regarding the FCA should be included in the text whether the instrument is standing on a bench or within a safety cabinet? Closed cabinet? The instrument location is important as information of the ventilation (air replaced XY times/hours). The information is important in order to valuate the results of the present study.
5. Methods: lack information when the measurement were done. At fixed times or random times both in research-  and clinical laboratory? The bacterial concentration in air is very closely associated to the number of staff at work as each person is calculated to release 5 CFU/bacteria per second to the environment.
6. The concentration of bacteria used in the FCA lacks (CFU/mL). The risk for environment contamination would probable increase at higher bacterial concentrationsversus low bacterial concentrations. Such comment should be discussed in the section discussion.
7. Section “Air sampling” Two types of media were used: 5% horse blood....
  I do recommend that this sentence is rewritten to pinpoint that culture of bacteria were done using 5% horse blood agar …. Information lacks for the supplier of the agar plates and if the MacConkey agar plates were incubated in CO2?  Especially if searching for S. pneumoniae.
8. Information of the supplier of the cotton swabs lacks as the size of the area that was swabbed by the cotton swabs? Bacterial content in the swabs is probably related to the area swabbed. Was the collection of specimens standardized?
9. MALDITOF. Definition of the “cut off” for correct / acceptable species identification lacks.
10. Headings of Table 1A, 1B, Table 2 has to be improved. Information regarding from which agar plates used should be presented in the section methods and omitted in the table headings.
  Table 1A, “Preparation bench” are “S. warneri, S. caprae”  and “S. xylosis, S. equus???” on the same lane and not consistent to the presentation of other species.
  Table 1B could be omitted since it does not contribute to the message in the study.
11. Figure 1, text has to be improved. Here, the comment “rises” should be omitted in text. The abbreviations “AFC” and “PB” should explained in the figure text.
  Figure 2, Figure text has to be rewritten, I recommend that either one of the terms “during use” or “before pre clean” is deleted or rewritten as “during use (pre-clean). There are no data presented “during use”, only pre-clean (=during use?) and post clean. Also, when (minutes) after cleaning was the specimens collected, this information should be included in the section “Material and methods”
12. The inclusion of a “clinical” laboratory” does contribute to the risk analysis for exposure of potential pathogens to the environment. The inclusion of av microbiological laboratory only serve as “control” to the microorganisms present in the research laboratory (FCA). The bacteria in the environment is as expected however gramnegative and grampositive bacteria were only present in the research unit which might need to be commented in the section “Discussion”.
13. Conclusion. The authors state that FCA do not pose a risk for FCA staff to pose a risk for FCA staff to be exposed to K pneumoniae, B thailandensis and S pneumoniae. I partly agree to the conclusion based on the results from the present study. The present study is small and further studies are warranted to confirm the present results.

 

 Conclusion: The risk for staff exposed to pathogen microorganisms during work is an important issue which is examined in the present study. Overall few studies are found regarding this subject. The present study is well done but more information has to included in text, see comments above.

 I recommend that the data from the clinical laboratory is excluded or put in as a text in the manuscript according the aim of the present study as I hardly find it to contribute to the risk assessment associated to the flow cytometry analysis.

 The article could be accepted after a major revision

I have read this submission. I believe that I have an appropriate level of expertise to confirm that it is of an acceptable scientific standard, however I have significant reservations, as outlined above.

# Response

**Published:** 2018-02-05

**Authors:** Timothy Inglis

**Reviewer**: The topic of the study is important in clinical laboratories regarding the risk for the staff to be exposed to harmful bacteria during their daily work. In this case risk for exposure to harmful bacteria during antimicrobial susceptibility test performed using a flow cell cytometric analysis. The method using “flow cytometric analysis” (FCA) is a relative new technique used in clinical laboratories and an expanding method I clinical microbiology.

I do have some major and minor comment to the present manuscript that has to be taken in consideration prior to acceptance of the manuscript.

**1. **The title of the manuscript do not clearly describe the intention of the present study. I would suggest that the title adjusted or rewritten to the message of the the present study.

- **Response**.The title has been amended to more accurately reflect the revised content.

**2. **Abstract: lack of “Aim(s)” in the text.

- **Response**. We have clarified text in the Title, Abstract, Introduction and Discussion to reflect that detection of viable aerosolized target bacteria before, during or after use of the acoustic-assisted flow cytometer as the aim of the study.

**3. **The authors includes a clinical laboratory (CL)  that do not use the FCA technique. However the bacterial concentration in air is measured and compared with that in the research laboratory using the FCA technique. However the design of the two laboratories (research- and clinical laboratory) are very different in construction and size (square meters) and if compared the number of staff/m2 should be presented.The clinical laboratory was included just to compare the distribution of bacteria in air with air the research laboratory which is of secondary interest as a risk assessment since the FCA instrument was not used in the clinical laboratory.

- **Response. **The inclusion of the nearby clinical laboratory as a sampling site has been more clearly justified. The following text has been added to the Methods, Laboratory Locations section:
  The clinical laboratory was included for comparative purposes. While the study aimed to determine if aerosolized viable target bacteria ( *B. thailandensis, K. pneumoniae *and *S. pneumoniae*) could be detected in air in the research laboratory, the likelihood was that other microorganisms, commonly present in indoor air, would be detected. Published data regarding the range and concentration of bacteria present in air in microbiology laboratories, either research or clinical, are scarce. Without data for comparison, data on the viable bacteria detected in air samples from the research laboratory would be entirely without context. Work practices in the clinical laboratory are designed to maintain a safe working environment and the background level of viable bacteria detected in clinical laboratory air could therefore serve as a proxy indicator of the acceptable level of viable bacteria in air in the research laboratory.
  Since background microorganisms would be detected in the air and, in an a priori effort to put them in some context, we sampled a non-flow cytometer site within the same research laboratory (preparation bench) and a non-flow cytometry laboratory (clinical laboratory) that handles numerous human bacterial pathogens using standard diagnostic microbiological techniques and biosafety risk management procedures.

**4. **Regarding the research laboratory information regarding the FCA should be included in the text whether the instrument is standing on a bench or within a safety cabinet? Closed cabinet? The instrument location is important as information of the ventilation (air replaced XY times/hours). The information is important in order to valuate the results of the present study.

- **Response**. Information about the position of the flow cytometer (on the bench, outside a safety cabinet) has been added. Approximate area and volume of the spaces has also been added. Unfortunately, data on the rate of air replacement were not available for either laboratory location.

**5. **Methods: lack information when the measurement were done. At fixed times or random times both in research-  and clinical laboratory? The bacterial concentration in air is very closely associated to the number of staff at work as each person is calculated to release 5 CFU/bacteria per second to the environment.

- **Response**. All the information about sampling times, sites and laboratory locations is given in the data file uploaded with this manuscript.
- It would be helpful to include the reference that calculates the release of bacteria into air from each person as 5 CFU of bacteria per second. We would be grateful if the reviewer could let us know the reference.

**6. **The concentration of bacteria used in the FCA lacks (CFU/mL). The risk for environment contamination would probable increase at higher bacterial concentrationsversus low bacterial concentrations. Such comment should be discussed in the section discussion.

- **Response**. This information has been added to the manuscript:
  For comparison, the second sampling site was on the preparation bench behind the operator approximately 2.5 m from the flow cytometer where 1 ml samples from bacterial cultures (generally 20-30 ml in trypticase soya broth or Mueller Hinton broth) were washed by centrifugation, mixed, diluted and further handled prior to analysis on the flow cytometer. In general, samples analyzed on the cytometer contained approximately 10 ^6^ bacteria/ml or less. During data acquisition for FAST assays, up to 12 samples were analyzed per bacterium. Sample acquisition halted after 1-3 minutes, and each FAST sample was acquired in technical triplicate.

**7. **Section “Air sampling” Two types of media were used: 5% horse blood....I do recommend that this sentence is rewritten to pinpoint that culture of bacteria were done using 5% horse blood agar …. Information lacks for the supplier of the agar plates and if the MacConkey agar plates were incubated in CO2?  Especially if searching for S. pneumoniae.

- **Response**. This information has been added to the manuscript:
  Two types of media were used: 5% horse blood agar (HBA) and MacConkey agar (MAC) (both supplied by PathWest Laboratory Medicine WA, Mt Claremont WA, Australia). HBA was included as a non-selective medium intended to allow the growth of any of the three target bacteria. MAC was included to minimize the growth of background non-target microorganisms (predicted to be mostly fungi or Gram positive bacteria) while still allowing the growth of B. thailandensis or K. pneumoniae. Samples taken on HBA while S. pneumoniae was being used in the research laboratory were incubated in the presence of 5% CO _2_. MAC plates were never incubated in CO _2_. All plates were incubated aerobically at 35°C. Colony forming units on both types of solid media were recorded after 24 hr incubation and expressed as CFU/1000 L air. Positive growth controls to confirm the ability of each of the three target bacteria to grow on the media under the chosen incubation conditions were performed.

**8. **Information of the supplier of the cotton swabs lacks as the size of the area that was swabbed by the cotton swabs? Bacterial content in the swabs is probably related to the area swabbed. Was the collection of specimens standardized?

- **Response**. Details of the swabs and the collection method have been added:
  Swabs (peel pouch Dryswab™ catalogue number MW112; Medical Wire and Equipment Company, Corsham, Wiltshire, England) were immersed in sterile 0.9% normal saline before rubbing vigorously over a 2.5 cm diameter circular area for 1.5 minutes in a spiral motion beginning at the center and rotating the swab continuously. Swabs were inoculated onto HBA first and then MAC using opposite sides of the swab for each plate. Swabs were collected at each site on at least two occasions.Incubation conditions were as previously described for air samples. Colony forming units on both types of solid media were recorded after 24 hr incubation and expressed as CFU/swab.

**9. **MALDITOF. Definition of the “cut off” for correct / acceptable species identification lacks.

- **Response**. The species cut-off for MALDI-TOF was >2.00. The genus level threshold was set at 1.80. This information has been added to the manuscript in the Bacterial identification section.

**10. **Headings of Table 1A, 1B, Table 2 has to be improved. Information regarding from which agar plates used should be presented in the section methods and omitted in the table headings.Table 1A, “Preparation bench” are “S. warneri, S. caprae”  and “S. xylosis, S. equus???” on the same lane and not consistent to the presentation of other species.Table 1B could be omitted since it does not contribute to the message in the study.

- **Response**. Headings and content for all tables have been amended. Table 1B has been retained but clarified.

**11. **Figure 1, text has to be improved. Here, the comment “rises” should be omitted in text. The abbreviations “AFC” and “PB” should explained in the figure text.Figure 2, Figure text has to be rewritten, I recommend that either one of the terms “during use” or “before pre clean” is deleted or rewritten as “during use (pre-clean). There are no data presented “during use”, only pre-clean (=during use?) and post clean. Also, when (minutes) after cleaning was the specimens collected, this information should be included in the section “Material and methods”

- **Response**. The Figure 1 legend text has been amended and the abbreviations addressed. It now reads:
  Figure 1. Airborne bacterial count detected on blood agar increases in the research lab over the course of the day, both beside the acoustic-assisted flow cytometer (AFC) and at the preparation bench (PB) (respective regression lines shown).
  The Figure 2 legend text has been amended. Previously it was: *Figure 2. Airborne bacterial count close to acoustic flow cytometer sample introduction port during use, before (pre clean) and after research laboratory cleaning Post clean), compared with the clinical microbiology laboratory. Pre clean research laboratory and post-clean median counts in close proximity to flow cytometer sample introduction port = 54 and 15.5 CFU/ 1000L, U = 2.5, p = 0.0090. *
  It now reads:
  Figure 2. Airborne bacterial counts from samples taken close to the acoustic flow cytometer sample introduction port during use, compared to counts from clinical laboratory samples. Data shown are for samples taken in the research laboratory before (pre-clean) and after (post-clean) interventional cleaning compared with samples from the clinical microbiology laboratory (CML) where no interventional cleaning was undertaken. Pre-clean and post-clean median counts from the research laboratory in close proximity to the flow cytometer sample introduction port = 54 and 15.5 CFU/ 1000L, U = 2.5, **p = 0.0090.
- Information about the timing of post-clean sampling has been added to the manuscript.

**12. Reviewer**. The inclusion of a “clinical” laboratory” does contribute to the risk analysis for exposure of potential pathogens to the environment. The inclusion of av microbiological laboratory only serve as “control” to the microorganisms present in the research laboratory (FCA). The bacteria in the environment is as expected however gramnegative and grampositive bacteria were only present in the research unit which might need to be commented in the section “Discussion”.

- **Response**. We agree with the reviewer that the inclusion of the clinical laboratory serves only to give some context to the microbial content of research laboratory air. The rationale for inclusion of the clinical laboratory has been expanded and clarified. See the information provided in response to review 2, point 1, bullet point 3.
- Gram negative bacteria were cultured in the air samples taken in the clinical laboratory. Clinical laboratory air samples on blood agar often had heavy growth and only the 6 commonest colony types were selected for identification. Gram negative bacteria did not feature amongst the 6 commonest colony types detected on blood agar (Table 1A). Clinical laboratory air samples on MacConkey agar grew Gram negative bacteria on three occasions. Attempts were made to identify all isolates growing on MacConkey agar but these were unidentifiable (see Table 1B). The text and the tables have been modified to clarify these points.

**13. **Conclusion. The authors state that FCA do not pose a risk for FCA staff to pose a risk for FCA staff to be exposed to K pneumoniae, B thailandensis and S pneumoniae. I partly agree to the conclusion based on the results from the present study. The present study is small and further studies are warranted to confirm the present results.

- **Response**. The outcomes of this study provided empirical reassurance to us that our current laboratory practices with these organisms do not pose a significant hazard to personnel. We agree that the study is small and that further studies are warranted to confirm and expand the present results.
- A section acknowledging the limitations of this study has been added to the manuscript.

**Reviewer: **Conclusion: The risk for staff exposed to pathogen microorganisms during work is an important issue which is examined in the present study. Overall few studies are found regarding this subject. The present study is well done but more information has to included in text, see comments above.

- **Response**. A significant quantity of additional information has been added throughout the manuscript.

**Reviewer**. I recommend that the data from the clinical laboratory is excluded or put in as a text in the manuscript according the aim of the present study as I hardly find it to contribute to the risk assessment associated to the flow cytometry analysis.

- **Response**. The Aim of the study has been clarified and the rationale for including the clinical laboratory as a comparator has been more clearly elaborated. Given the information vacuum around the microbial content of indoor air in microbiology laboratories of either persuasion, clinical or research, we respectfully disagree with the reviewer on the value of retaining the data from the clinical laboratory.

**Reviewer**. The article could be accepted after a major revision

**Response**. We have addressed all of this reviewer’s comments as well as all the comments from the other 2 reviews. We believe this constitutes a major revision and that the manuscript should now be acceptable.

We would be grateful if the reviewer could let us know the reference for the 5CFU of bacteria released per person per second.
